# Supplementary material for: Count your bits: fingerprint benchmarking to assess broad chemical space representation
Source: J Cheminform. 2026 Jun 30;18:83. doi: 10.1186/s13321-026-01245-y (PMC13317254; doi:10.1186/s13321-026-01245-y)
Supplement: Supplementary file 1 — Supplementary Material 1. [file 13321_2026_1245_MOESM1_ESM.pdf]

Supplemental material

Count your bits: fingerprint benchmarking to assess broad chemical space representation

Florian Huber, Julian Pollmann

Contents

Supplemental material 1
Basic dataset statistics 2
Number of non-zero bits for different fingerprint variants 3
Fingerprint Comparisons Reveal Divergent Perspectives on Molecular Similarity 4
Fingerprint bit occupation when compared to more compressed 1024-bit variants 7
Examples of high mass difference fingerprint duplicates (MACCS) 8
Examples of high mass difference duplicates - RDKit binary (4096 bits) 9
Mass-sensitivity of different similarity measures 10
Top-10 ranking overlaps between different fingerprint types and variants 11
Activity prediction vs. virtual screening experiment 12
Subclass prediction task (120-subclasses dataset) 15
Subclass neighborhood consistency (25-subclasses dataset) 16
Exploring individual bit counts and weighing options 19
Additional UMAP visualizations (biostructures dataset, using cuml & cosine) 22
Additional UMAP visualizations (ms2structures dataset) 23
References 23

Basic dataset statistics

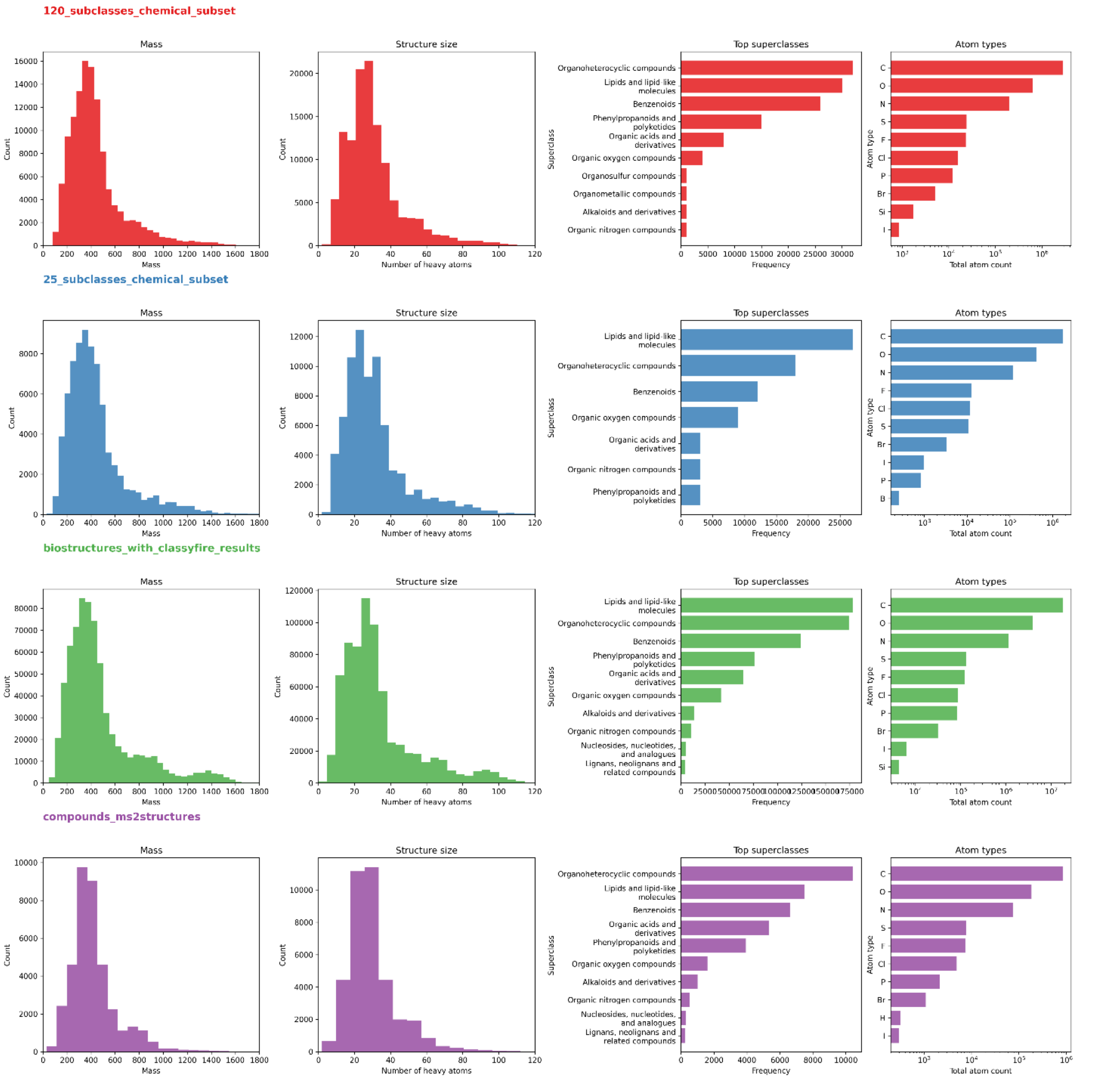

Figure 1 Basic statistics of the four main datasets used in this study showing the molecular mass distribution (left column), the distribution of the number of heavy atoms per molecule (second column from left), the most common Classyfire superclasses (second column from the right), and the most common elements (right column).

Number of non-zero bits for different fingerprint variants

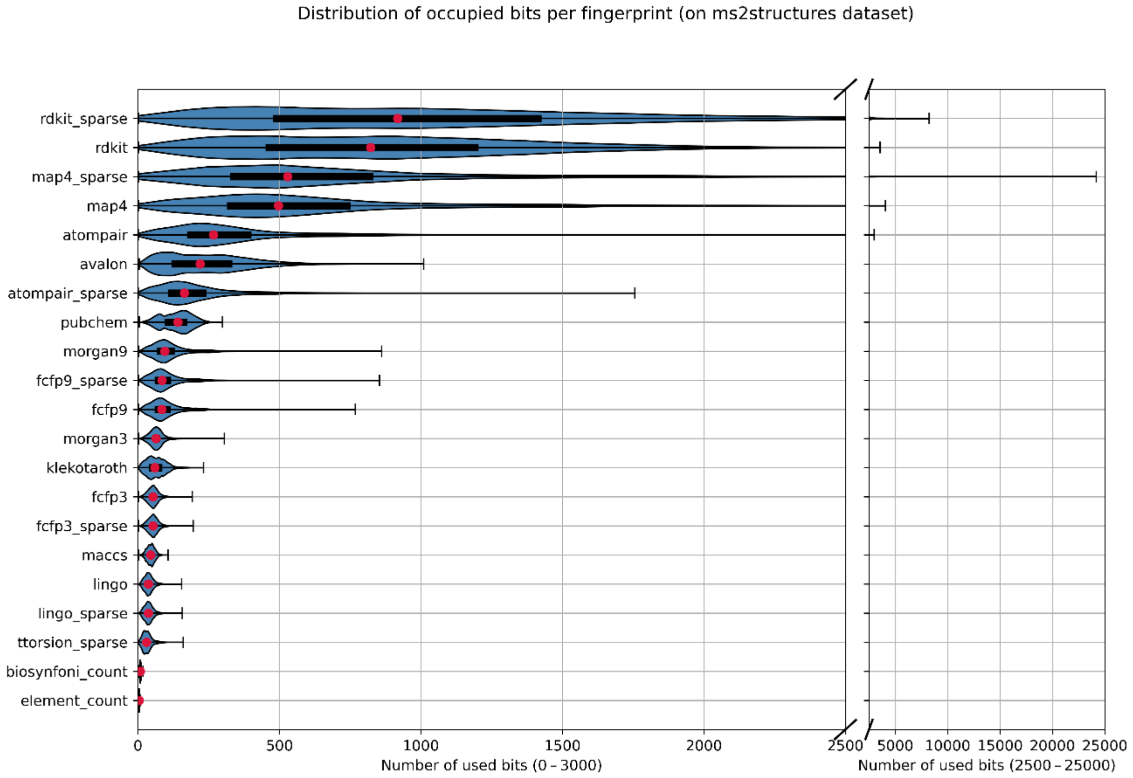

Figure 2 Distributions showing the number of non-zero bits over the entire ms2structures dataset. The subplot on the right shows the long tail of some of the distributions towards reflecting few compounds which led to very high numbers of occupied bits. The unfolded MAP4 implementation, for instance, resulted in fingerprints of up to 24,183 bits.

Fingerprint Comparisons Reveal Divergent Perspectives on Molecular Similarity

Different fingerprint representations capture distinct aspects of molecular structure, and these differences are reflected in their similarity score distributions. To give a first intuition, we here compare RDKit fingerprints and Morgan fingerprints, across the 37,811 compounds in the **ms2structures dataset**. Although both fingerprint types are commonly employed in tasks such as compound ranking, their underlying algorithms and bit occupancy rates differ noticeably, leading to different interpretations of what it means for two molecules to be similar.

Due to its higher bit occupation rates, the default RDKit fingerprints generally produce higher Tanimoto similarity scores compared to Morgan-3 fingerprints. To enable a more meaningful comparison, the raw similarity scores were converted into percentile values ranging from 0% to 100% based on all unique compound pairs (see methods). This scaling allowed us to directly contrast how each fingerprint representation ranks molecular similarity across the entire dataset.

Comparison 1: Morgan-3 Binary vs. RDKit Binary Fingerprints
When comparing Tanimoto similarity scores computed from Morgan-3 binary (4096-bit) fingerprints with those derived from RDKit binary fingerprints of the same bit length, substantial differences in their percentile distributions were observed. Notably, a significant proportion of compound pairs, approximately 43.41%, were consistently assigned to the bottom 60% of similarity by both scoring methods.
In contrast, only a smaller fraction of pairs (0.42%) was jointly classified within the top 1% of similarity. Moreover, there were striking discrepancies for certain compound pairs. For instance, over 200,000 pairs (0.04% of all unique pairs) were placed in the top 1% by RDKit fingerprints while being relegated to the bottom 60% by Morgan-3 fingerprints.
Conversely, more than 900,000 pairs (0.13%) were ranked in the top 1% by Morgan-3 fingerprints but fell into the bottom 60% based on RDKit scores (Figure 3). These results underline that even when using the same Tanimoto metric, the two fingerprint types emphasize different molecular features, resulting in divergent similarity assessments.

Comparison 2: Morgan-3 Binary vs. Morgan-3 Count
To further probe the influence of fingerprint representation, we compared Tanimoto scores computed from Morgan-3 binary fingerprints with Tanimoto similarity scores derived from Morgan-3 count-based fingerprints. In this pairing, both methods are based on the same underlying fingerprint type, yet the representation (binary versus count) introduces noteworthy differences.
The analysis revealed that about 50.42% of compound pairs were consistently ranked in the bottom 60% by both metrics, while 0.77% of pairs were jointly assigned to the top 1%. Although the overall agreement between the two metrics is higher than in the previous comparison, notable differences persist for certain compound pairs.
The count-based fingerprints offer the advantage of recognizing repeated occurrences of motifs or substructures, as exemplified in an example pair in Figure 3, providing a more nuanced view of molecular similarity. However, this sensitivity can also be a drawback, as it may overemphasize the contribution of common substructures, such as extended carbon chains (e.g., CC chains) leading to disproportionately high similarity scores in some cases.

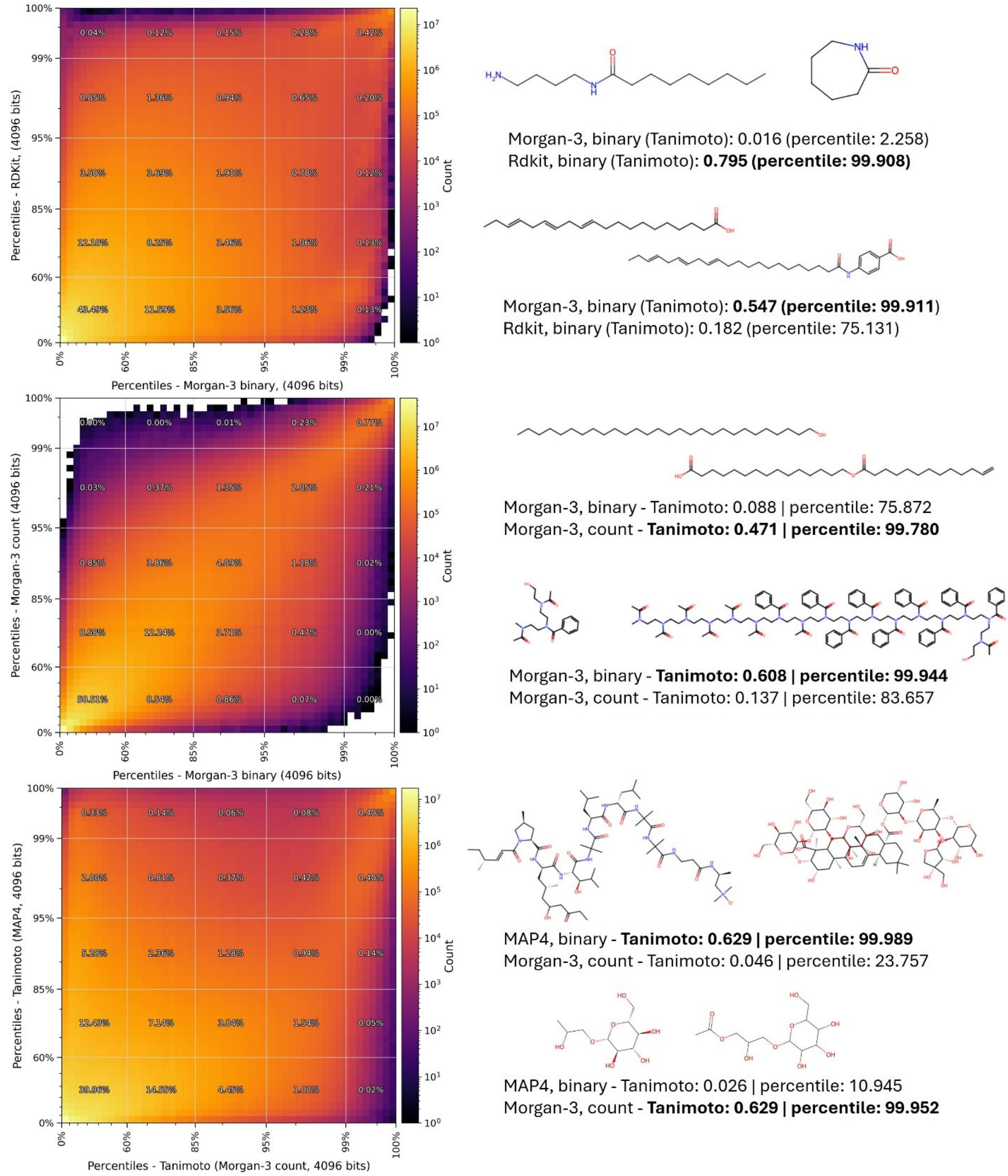

Figure 3 Comparison of all pairwise similarities for the ms2structures dataset (37,811 compounds) using various fingerprints. ***(top row)*** *Tanimoto scores computed from RDKit fingerprints and Morgan-3 binary fingerprints.* ***(center row)*** *Tanimoto scores computed from Morgan-3 binary fingerprints and from Morgan-3 count-based fingerprints.* ***(bottom row)*** *Tanimoto scores were computed from Morgan-3 count fingerprints and from MAP4 fingerprints. All fingerprints were 4096-bit vectors. Example pairs with drastic score discrepancies are highlighted on the right.*

Comparison 3: MAP4 vs. Morgan-3 Count

The third comparison involves Tanimoto similarity scores computed using MAP4 fingerprints and from Morgan-3 count-based fingerprints, with both representations using 4096-bit vectors. As illustrated in Figure 3 (bottom row), the overall correspondence between these two metrics is less pronounced than in the previous comparisons. Only 39.97% of compound pairs fall into the bottom 60% for both metrics, and just 0.39% are classified in the top 1% by both methods. Notably, MAP4 exhibits the highest bit occupation among the fingerprints evaluated, a characteristic that appears to lead to a substantial number of bit collisions, particularly for larger molecules. This increased collision rate manifests in a sizable subset of cases: approximately 0.33% of pairs are ranked in the top 1% for MAP4 while simultaneously being assigned to the bottom 60% according to the Tanimoto score on Morgan-3 count fingerprints.

In addition, a smaller group of pairs is observed in the opposite corner of the score distribution, where the Morgan-based count fingerprints place them in the top 1% while MAP4 assigns them to the bottom 60%. We noted that those pairs are usually clear cases of unambiguous false similarity assignments by MAP4 as indicated by the two example pairs in Figure 3.

Fingerprint bit occupation when compared to more compressed 1024-bit variants

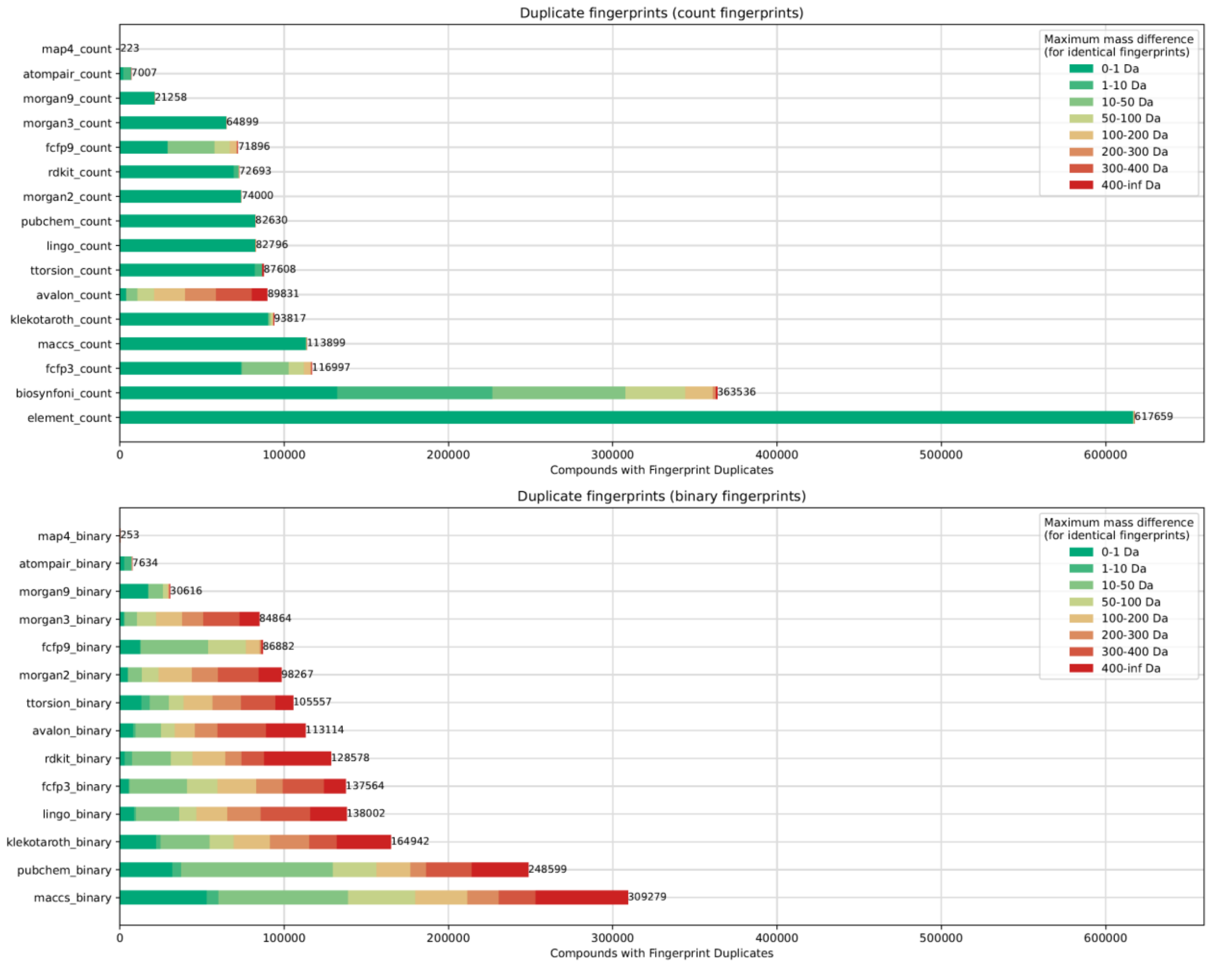

Figure 4 For different molecular fingerprints, all duplicates in a set of 718,067 unique compounds (biomolecular structures dataset) were counted. For each duplicate fingerprint, we computed the maximum mass difference among compounds sharing that fingerprint which is colored along different bins ranging from 0-1Da mass difference up to mass differences >= 400 Da. Here, unlike in the main article, we also included Biosynfoni, and, as a baseline, a simple element count vector.

Examples of high mass difference fingerprint duplicates (MACCS)

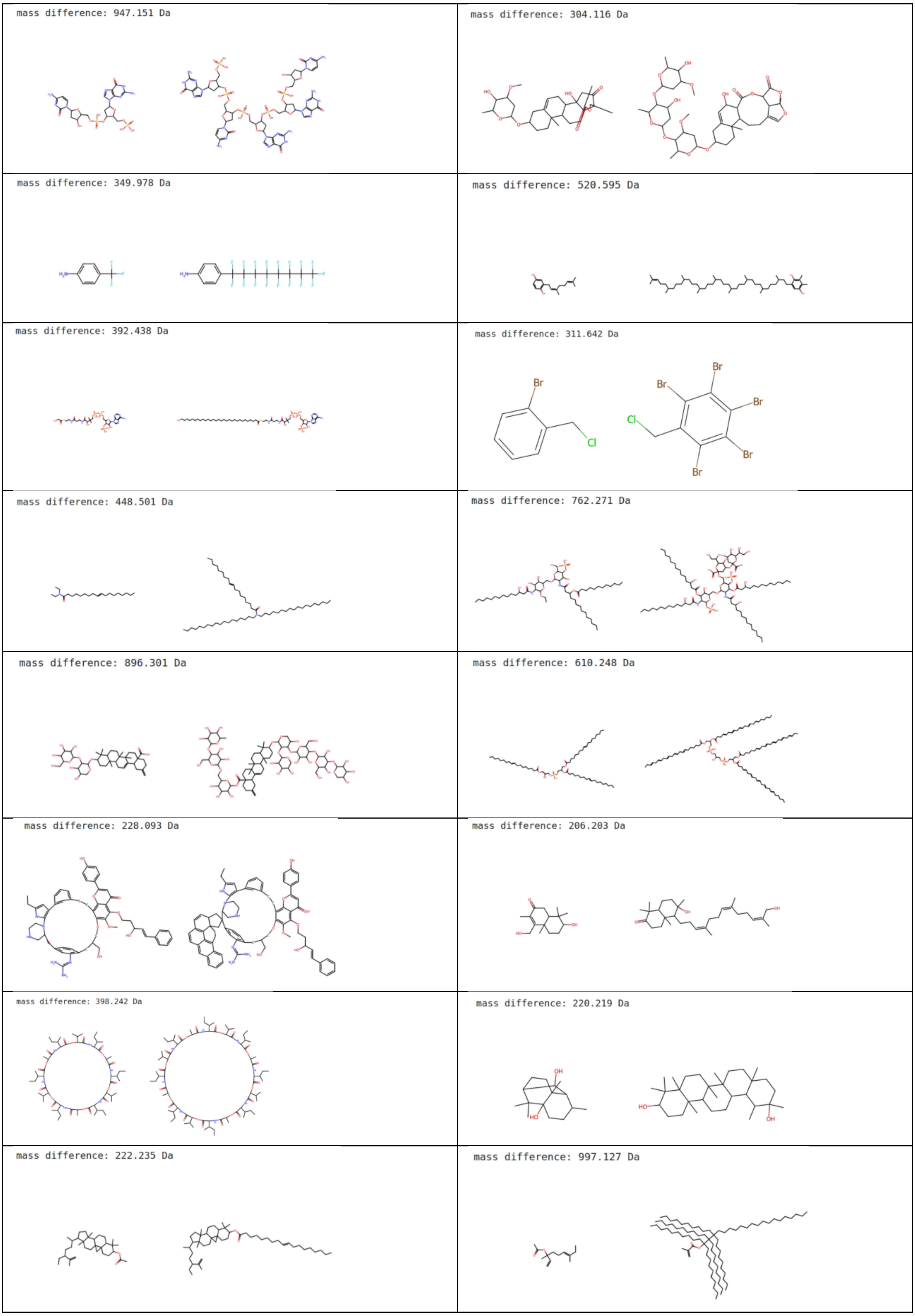

Examples of high mass difference duplicates- RDKit binary (4096 bits)

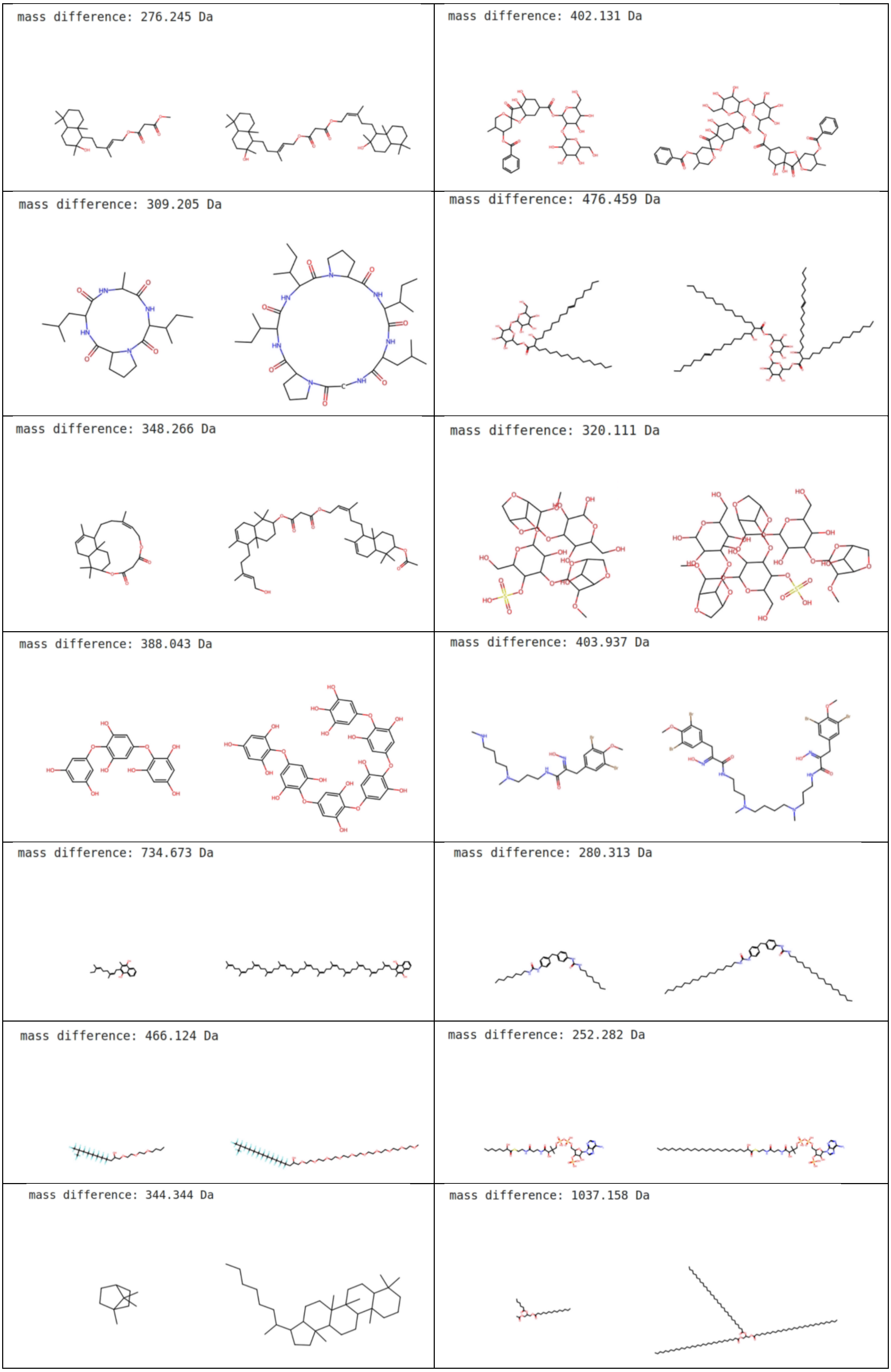

Mass-sensitivity of different similarity measures

In the main article we used a percentile scaling to better compare the different similarity scores. Here, just for comparison, we also display the overall similarity scores for all compounds <300Da and >500Da in the ms2structures dataset without the percentile scaling.

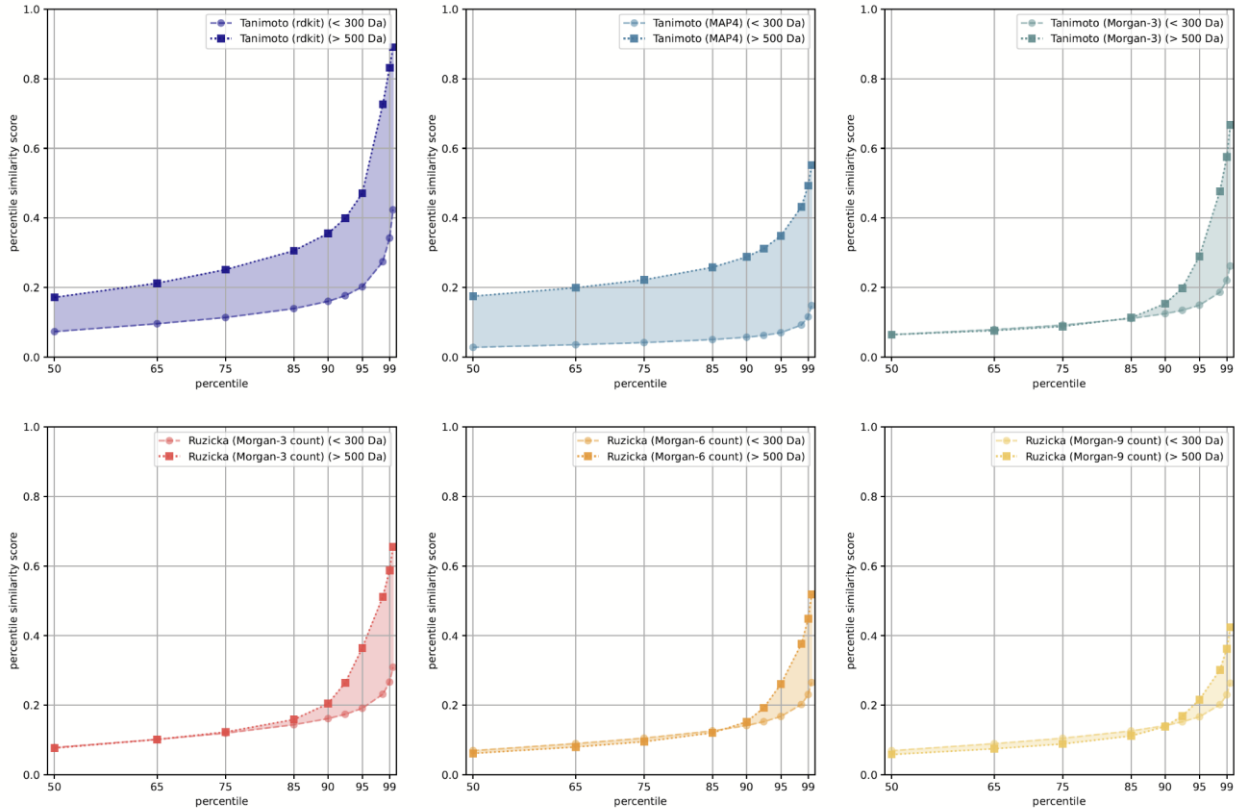

Figure 5 Fingerprint-based similarities are computed for about 36 million unique pairs between small compounds (< 300 Da) or between larger compounds (> 500 Da). The similarity scores of increasing percentiles are plotted for both the small and larger molecules ranging from 50% up to 99.5%. This is done for Tanimoto scores of rdkit fingerprints (top left), Tanimoto scores of MAP4 fingerprints (top, middle), Tanimoto scores of Morgan-3 binary fingerprints (top right) as well as Ruzicka scores of Morgan-3 to Morgan-9 count vectors (bottom row). All used fingerprints had 4096 bits.

Top-10 ranking overlaps between different fingerprint types and variants

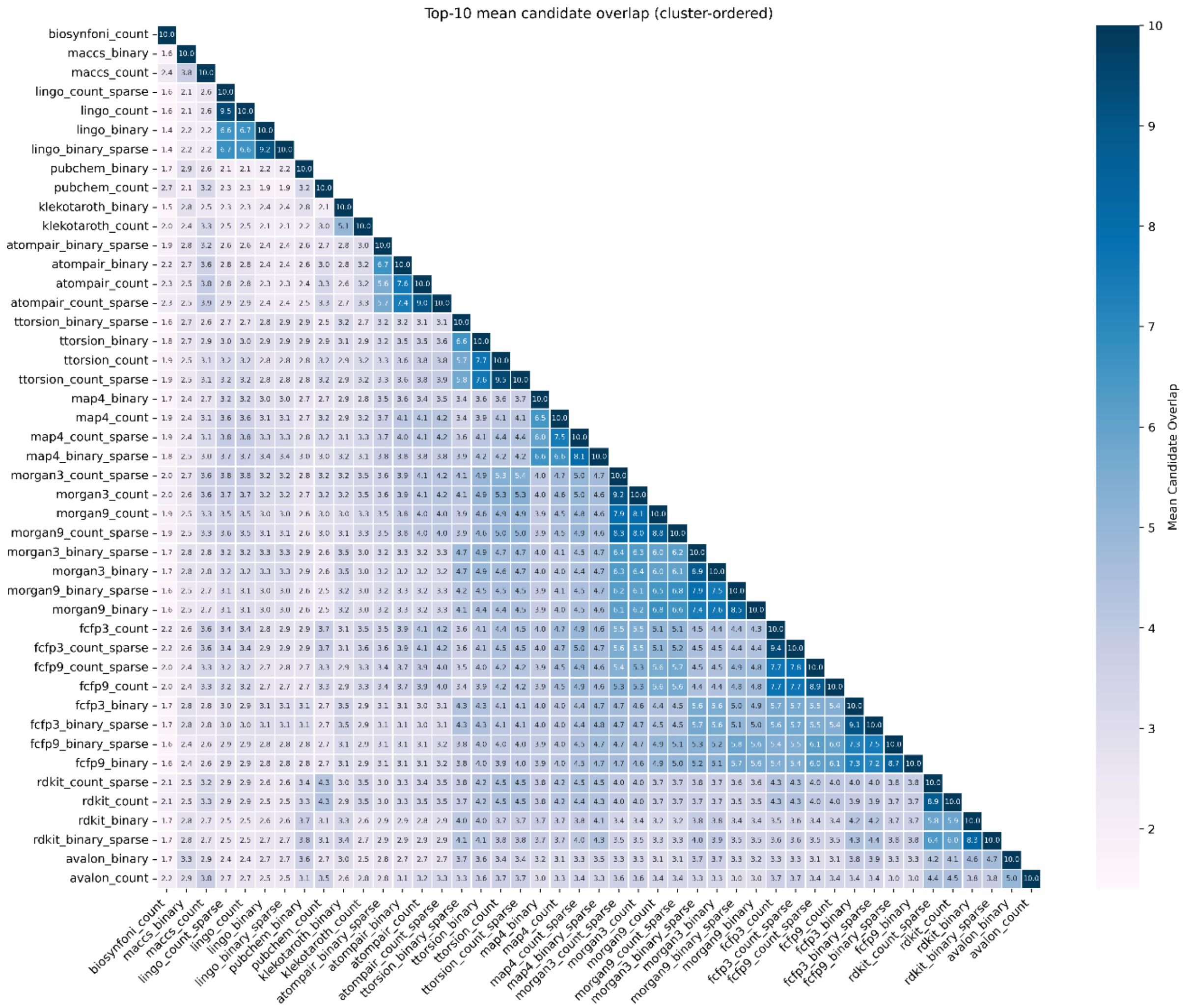

Figure 6 Heatmap to display all top-10 ranking overlaps between the different fingerprint types and variants. Those values were used to later compute a minimum spanning tree (see main article).

Activity prediction vs. virtual screening experiment

A frequently cited, and adapted, fingerprint evaluation method is the one by Riniker and Landrum [1].

In the context of our attempt to shed a rather broad perspective on fingerprint similarity, we contend that this ranking-based aggregation might introduce two potential issues, one with the aggregation of ranks and one with the underlying screening task. Aggregation of ranks is a commonly applied technique, mostly because it focuses on relative performance per query, is robust to metric outliers, and avoids unjust bias towards methods optimized on high-variance subsets [2]. But converting continuous performance differences into ordinal ranks can obscure the true magnitude of those differences. Even minimal differences may result in a rank change of one, while substantial differences may be treated equivalently if they occupy consecutive rank positions. And, the averaged ranks are not independent quantities but depend on the selection of competing scores.

Secondly, the ranking tasks in the mentioned studies [1], [3], [4] are geared towards detecting compounds with assigned biological activity within a large pool of decoys, thereby simulating the detection of active compounds. This, however, is not directly related to the conceptual design of most fingerprints, which are rather broad descriptors of overall molecular substructures.

We ran the ranking script from Orsi and Reymond [4] with various sparse Morgan-based fingerprints. Overall ranking performance on the datasets provided for small molecules remained best for the MAP4 fingerprints (see Figure S2 in supplemental material).

Since those mean ranking plots depend on the selection of scores and hence do not allow for a flexible, general benchmarking over many different scores and future adaptations, we here also compare several other performance metrics as used in [4] (Figure 6). Here, too, MAP4 frequently received the best average metrics, though not on all provided metrics.

Interestingly, we found that larger radius Morgan fingerprints, such as sparse log-count Morgan-9 fingerprints perform comparably well on the peptide task, being roughly on par with MAP4 for the mean rank (Figure 7) as well as the difference mean metrics (Figure 9).

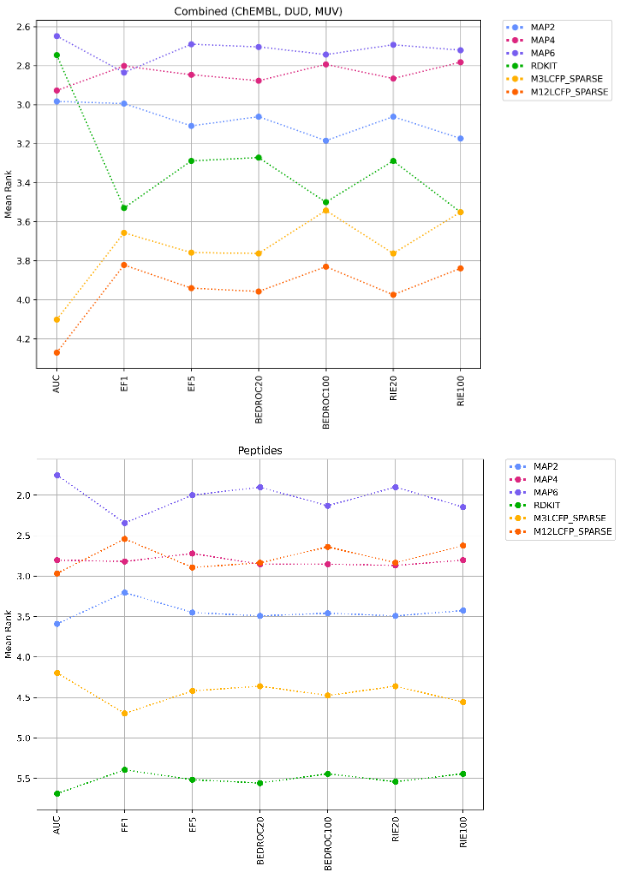

Figure 7 Mean ranks of fingerprints across all virtual screening datasets as provided in Orsi and Raymond (2024). Left: For the three datasets for small molecules (ChEMBL, DUD, MUV). Right: For the peptides datasets.

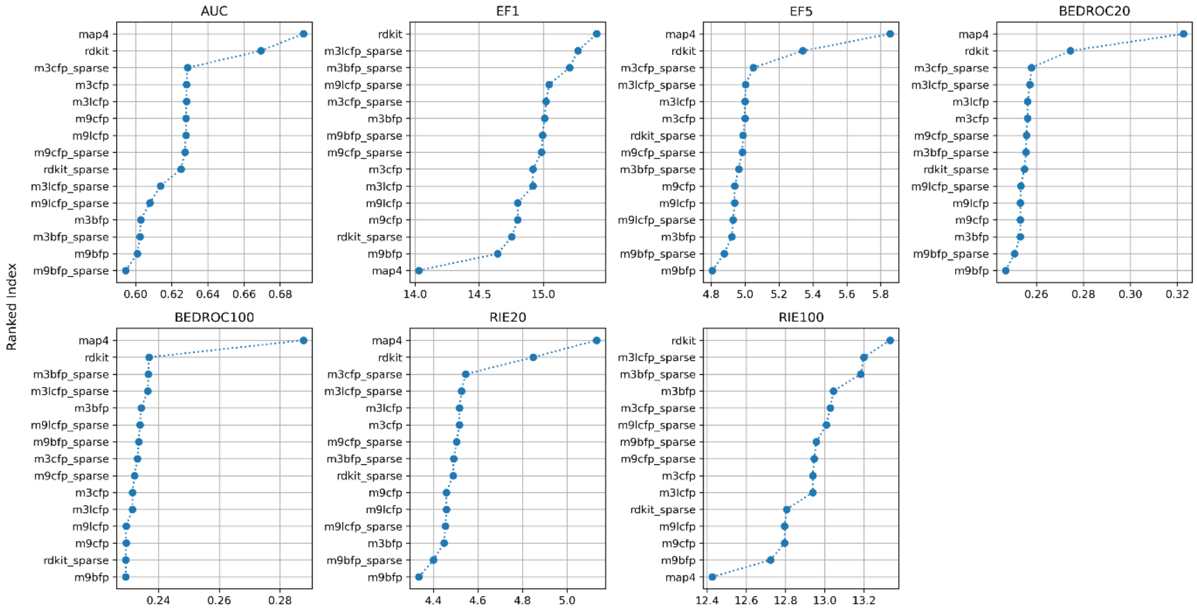

Figure 8 Mean scores of fingerprints across the 3 virtual screening datasets for ***small molecules (ChEMBL, DUD, MUV)*** *as provided in Orsi and Raymond (2024). For each subplot the respective values were ordered in descending order to allow identifying which fingerprints performed well or poorly according to various metrics.*

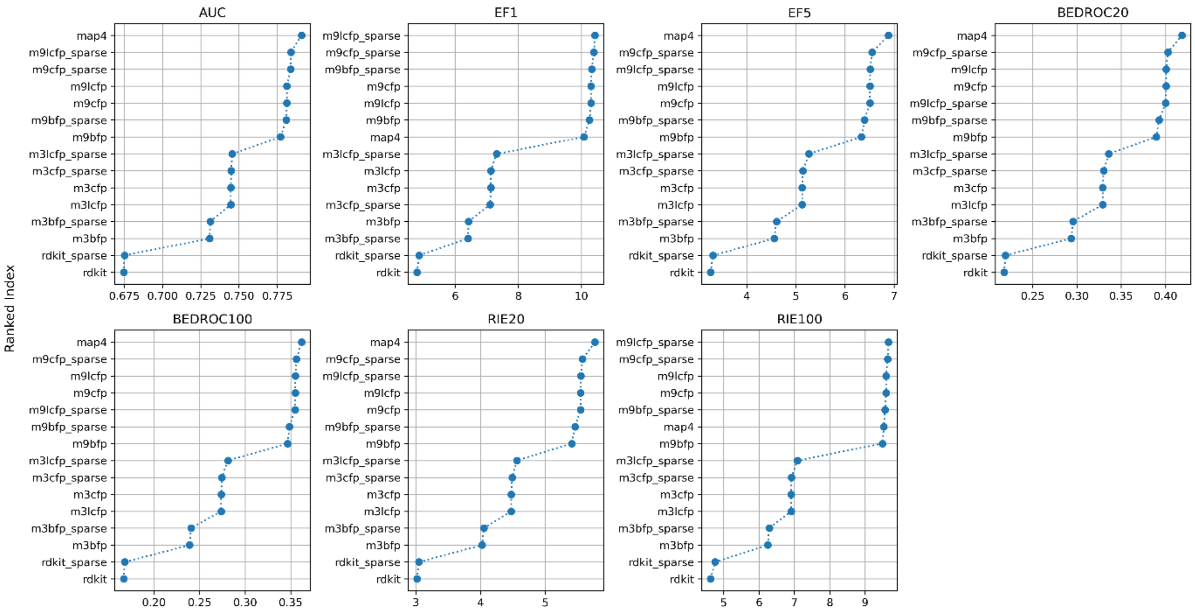

Figure 9 Mean scores of fingerprints across the 2 virtual screening datasets for ***peptides*** *as provided in Orsi and Raymond (2024). For each subplot the respective values were ordered in descending order to allow identifying which fingerprints performed well or poorly according to various metrics.*

Subclass prediction task (120-subclasses dataset)

In the main article, we did not include the results for Biosynfoni for the 120 subclass prediction task because it became clear, that the fingerprint was not providing enough information for the models to compete with the other, much larger, fingerprint types. We expect, that it might still perform much better on a subset of the subclasses we trained on, but we have not done any steps to evaluate this any further.

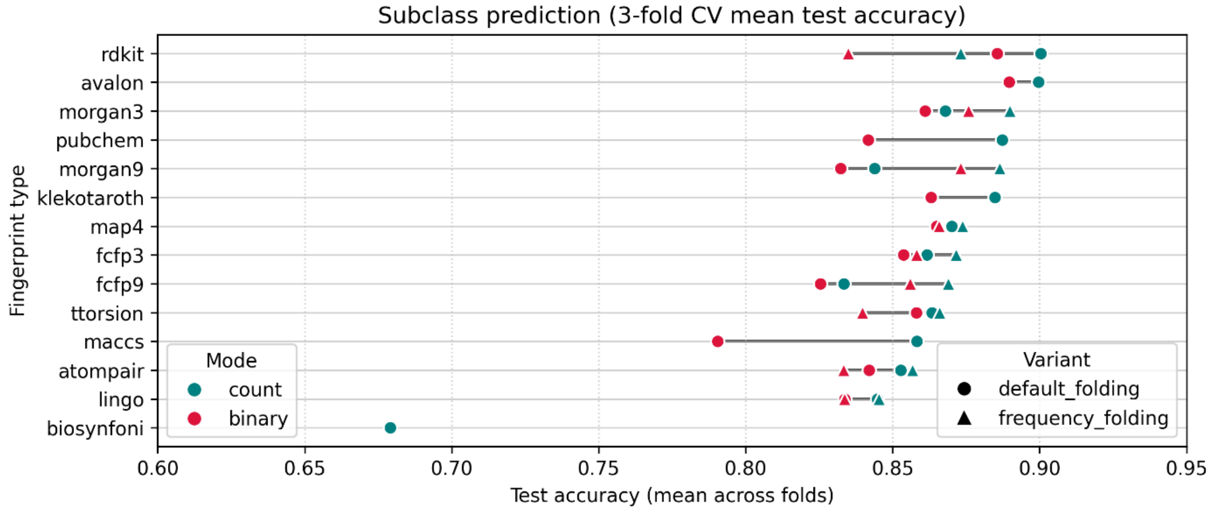

Figure 10 In the main article we excluded Biosynfoni because it could not compete with the other, larger, fingerprint types and variants.

As mentioned in the main article, our goal was not to train the most performant models possible. We used, on purpose, only a subset of the available data. And we also did not do very excessive parameter scans. So we expect that the actual accuracy ratings can be improved further by using more training data and spending more time to optimize model architecture and training procedure.

As a sanity check, however, we did train a much simpler architecture with only one hidden layer (3000 nodes). While some accuracy values changed slightly, the overall results were largely on par with the deeper model used in the main article.

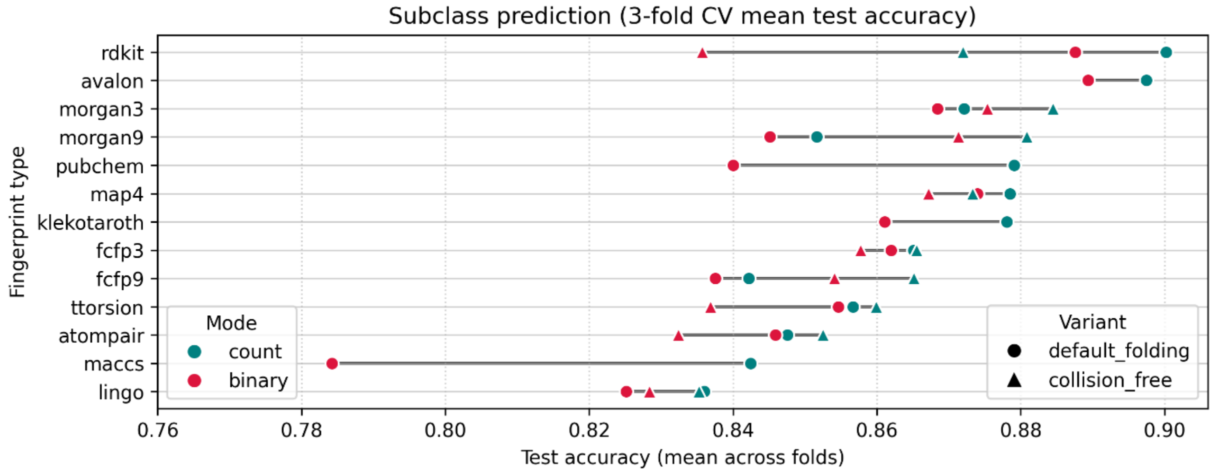

Subclass neighborhood consistency (25-subclasses dataset)

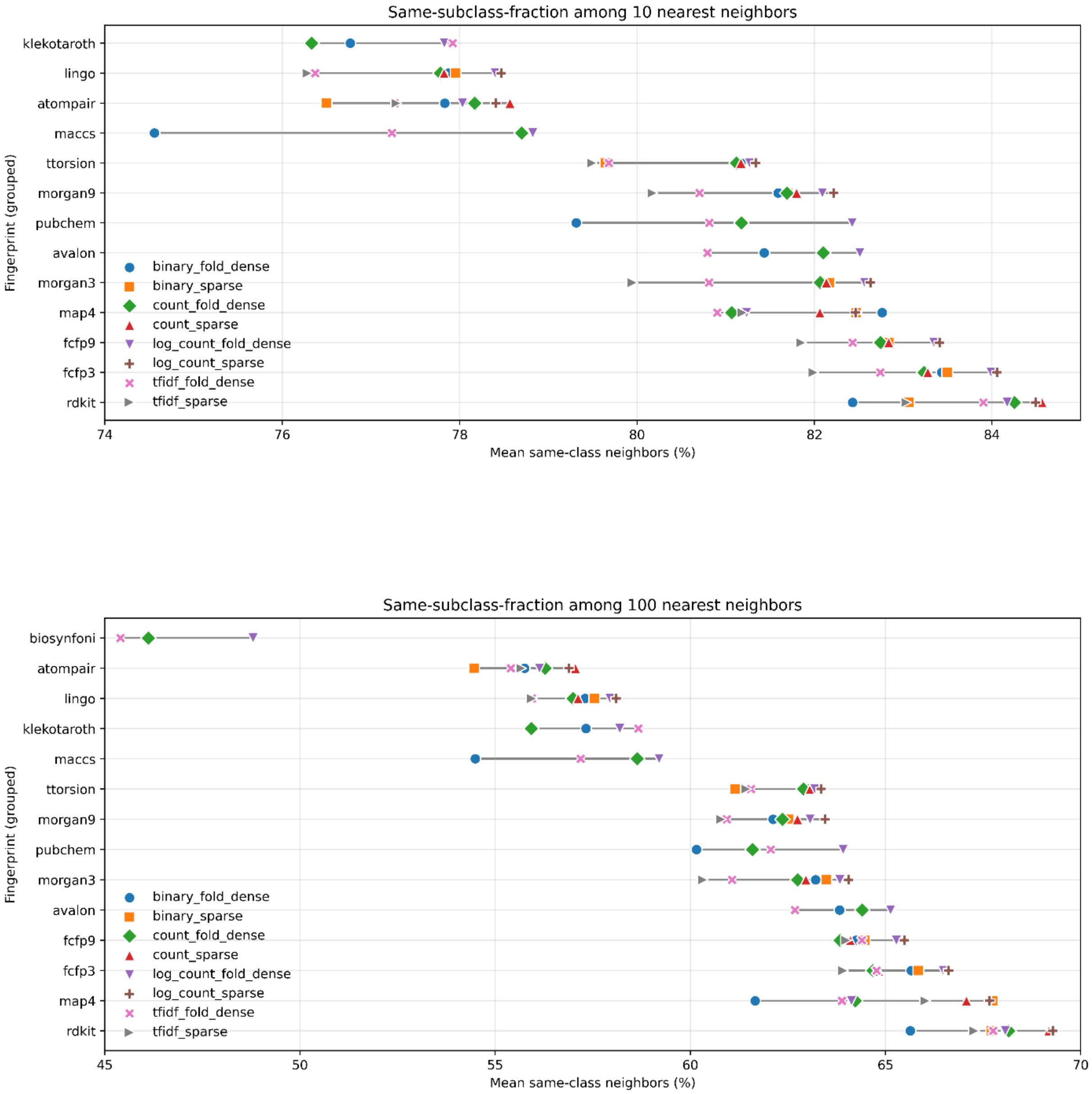

Figure 11 Plot of all fingerprint types, including Biosynfoni on the subclass consistency.

The following plot show the measured same-class consistency for each of the 25 selected subclasses.

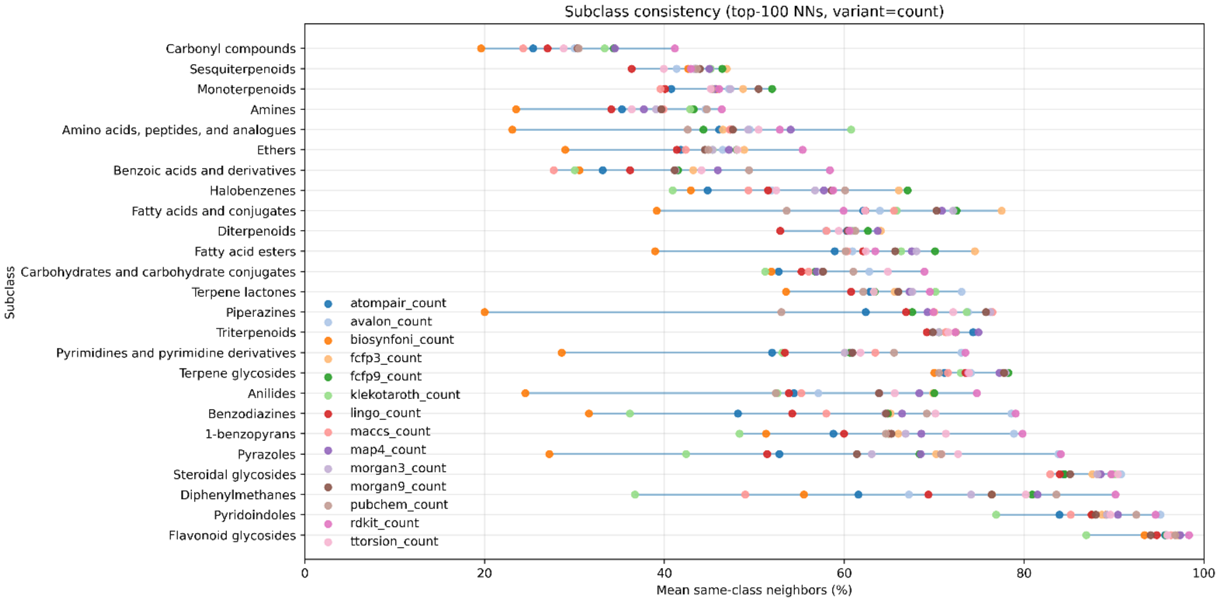
Subclass consistency (top-100 NNs, variant=count)
Carbonyl compounds
Sesquiterpenoids
Monoterpenoids
Amines
Amino acids, peptides, and analogues
Ethers
Benzoic acids and derivatives
Halobenzenes
Fatty acids and conjugates
Diterpenoids
Fatty acid esters
Carbohydrates and carbohydrate conjugates
Terpene lactones
Piperazines
Triterpenoids
Pyrimidines and pyrimidine derivatives
Terpene glycosides
Anilides
Benzodiazines
1-benzopyrans
Pyrazoles
Steroidal glycosides
Diphenylmethanes
Pyridoindoles
Flavonoid glycosides
Subclass
atompair_count
avalon_count
biosynfoni_count
fcfp3_count
fcfp9_count
klekotaroth_count
lingo_count
maccs_count
map4_count
morgan3_count
morgan9_count
pubchem_count
rdkit_count
ttorsion_count
0
20
40
60
80
100
Mean same-class neighbors (%)

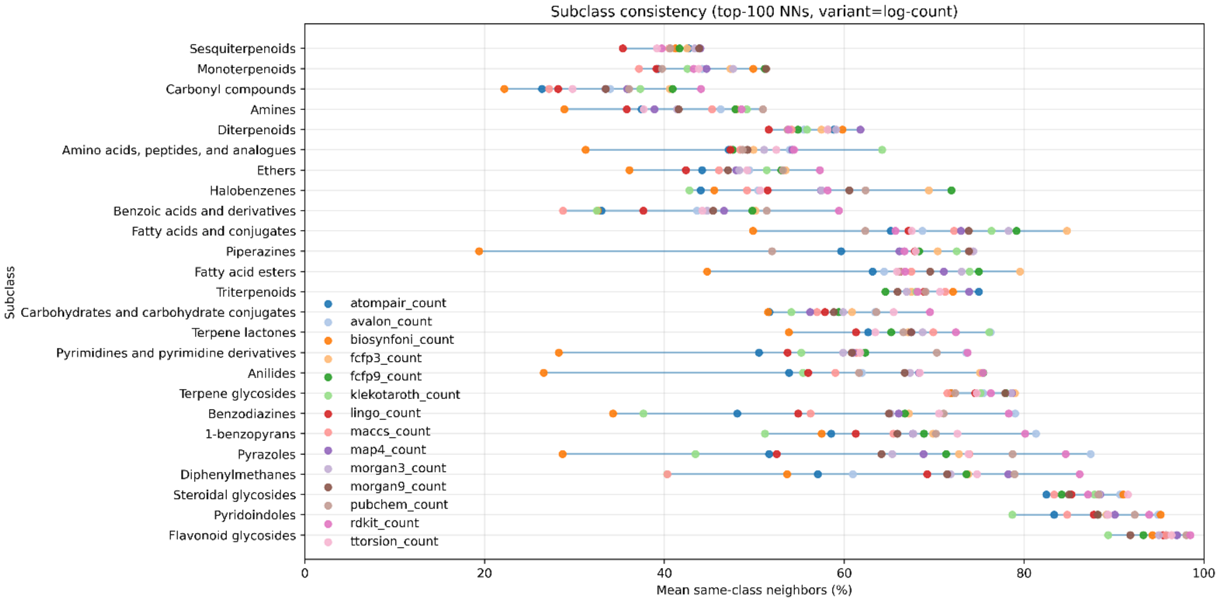
Subclass consistency (top-100 NNs, variant=log-count)
Sesquiterpenoids
Monoterpenoids
Carbonyl compounds
Amines
Diterpenoids
Amino acids, peptides, and analogues
Ethers
Halobenzenes
Benzoic acids and derivatives
Fatty acids and conjugates
Piperazines
Fatty acid esters
Triterpenoids
Carbohydrates and carbohydrate conjugates
Terpene lactones
Pyrimidines and pyrimidine derivatives
Anilides
Terpene glycosides
Benzodiazines
1-benzopyrans
Pyrazoles
Diphenylmethanes
Steroidal glycosides
Pyridoindoles
Flavonoid glycosides
Subclass
atompair_count
avalon_count
biosynfoni_count
fcfp3_count
fcfp9_count
klekotaroth_count
lingo_count
maccs_count
map4_count
morgan3_count
morgan9_count
pubchem_count
rdkit_count
ttorsion_count
0
20
40
60
80
100
Mean same-class neighbors (%)

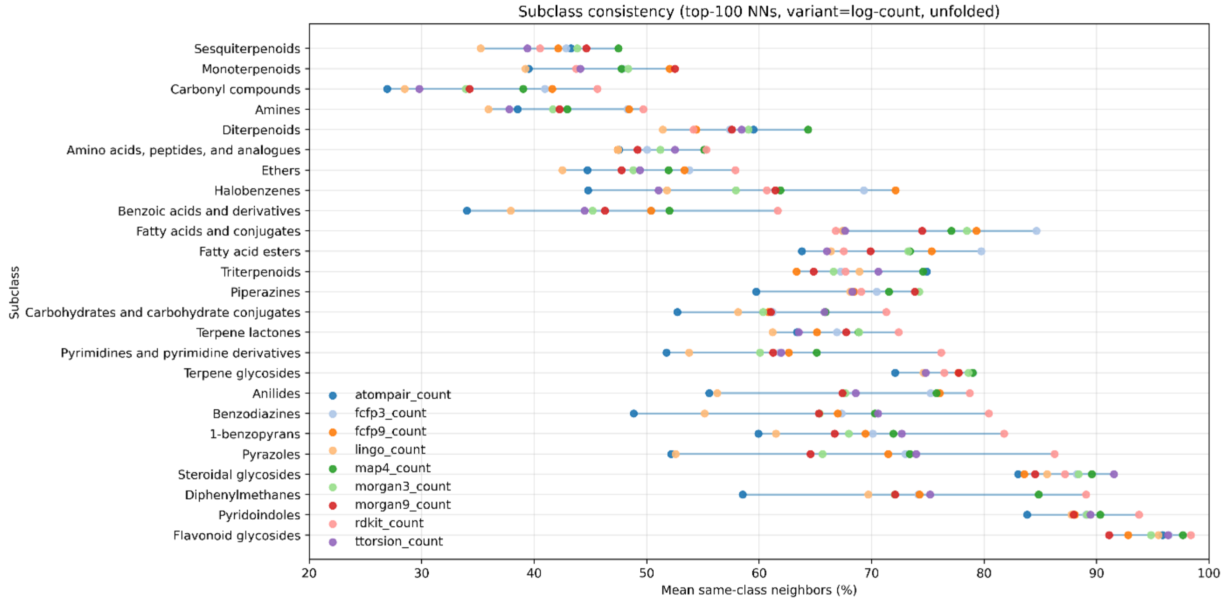
Subclass consistency (top-100 NNs, variant=log-count, unfolded)
Sesquiterpenoids
Monoterpenoids
Carbonyl compounds
Amines
Diterpenoids
Amino acids, peptides, and analogues
Ethers
Halobenzenes
Benzoic acids and derivatives
Fatty acids and conjugates
Fatty acid esters
Triterpenoids
Piperazines
Carbohydrates and carbohydrate conjugates
Terpene lactones
Pyrimidines and pyrimidine derivatives
Terpene glycosides
Anilides
Benzodiazines
1-benzopyrans
Pyrazoles
Steroidal glycosides
Diphenylmethanes
Pyridoindoles
Flavonoid glycosides
Subclass
atompair_count
fcfp3_count
fcfp9_count
lingo_count
map4_count
morgan3_count
morgan9_count
rdkit_count
ttorsion_count
20
30
40
50
60
70
80
90
100
Mean same-class neighbors (%)

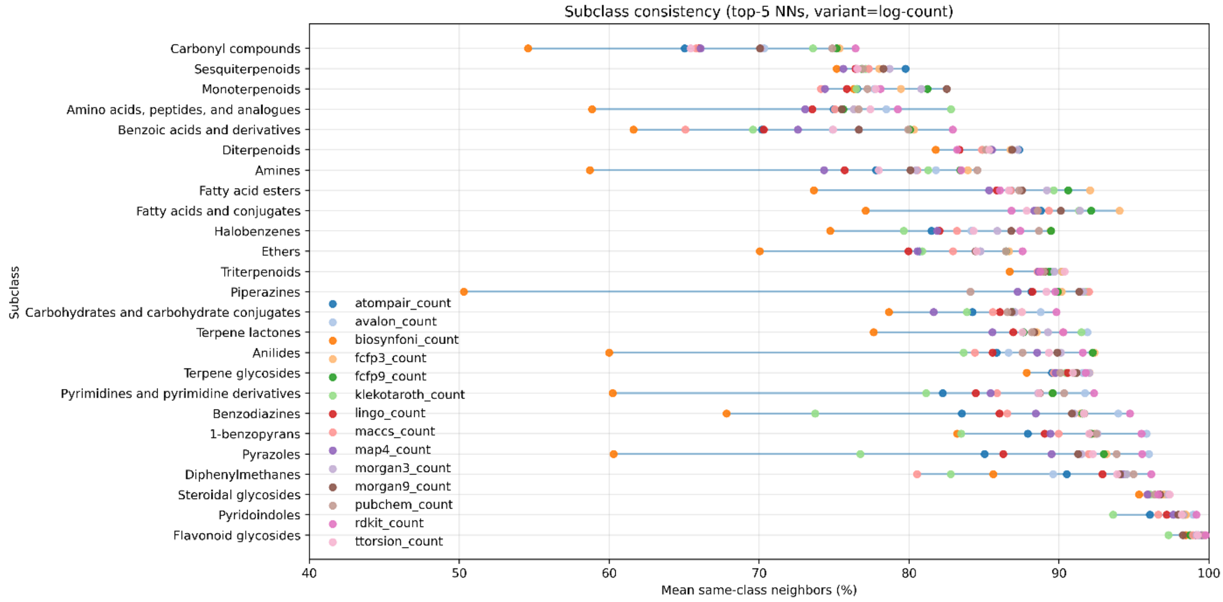
Subclass consistency (top-5 NNs, variant=log-count)
Carbonyl compounds
Sesquiterpenoids
Monoterpenoids
Amino acids, peptides, and analogues
Benzoic acids and derivatives
Diterpenoids
Amines
Fatty acid esters
Fatty acids and conjugates
Halobenzenes
Ethers
Triterpenoids
Piperazines
Carbohydrates and carbohydrate conjugates
Terpene lactones
Anilides
Terpene glycosides
Pyrimidines and pyrimidine derivatives
Benzodiazines
1-benzopyrans
Pyrazoles
Diphenylmethanes
Steroidal glycosides
Pyridoindoles
Flavonoid glycosides
Subclass
atompair_count
avalon_count
biosynfoni_count
fcfp3_count
fcfp9_count
klekotaroth_count
lingo_count
maccs_count
map4_count
morgan3_count
morgan9_count
pubchem_count
rdkit_count
ttorsion_count
40
50
60
70
80
90
100
Mean same-class neighbors (%)

Exploring individual bit counts and weighing options

The bit occupation distributions in shown in the main article (**Error! Reference source not found.**) displays that some fingerprint bits occur much more often than others. In the logic of both binary and count-based fingerprints, such as the RDKit and Morgan fingerprints, but also MAP4, all fingerprint bits are equally important. This means that for a similarity computation, the presence or absence of a very common substructure counts as much as the presence or absence of a rarely occurring substructure.

We weighted each fingerprint bit using the inverse document frequency (IDF, see Methods). This was done using sparse RDKit and Morgan fingerprints to avoid bit collisions. As expected, the most frequent substructures for Morgan fingerprints were of radius 0 or 1 and exclusively contained the atoms H, C, O, N (Figure 12). The most frequent substructure, an oxygen atom with a double bond, was found in about 84% of all fingerprints in the ms2structures dataset and in about 81% of all fingerprints in the biostructures dataset (Figure 13). RDKit fingerprints contain many more substructures that occur in a large fraction of all molecules, in agreement with **Error! Reference source not found.** (main article). The most frequent substructures here are C-C sequences of various lengths as well as other trivial motifs like C-O or C=O (Figure 14).

On both the ms2structures and the biostructures dataset, we computed sparse binary Morgan-3 fingerprints for all compounds. The total number of occurrences of each bit (=substructure) across the respective dataset was then taken to compute the IDF weights for each bit.

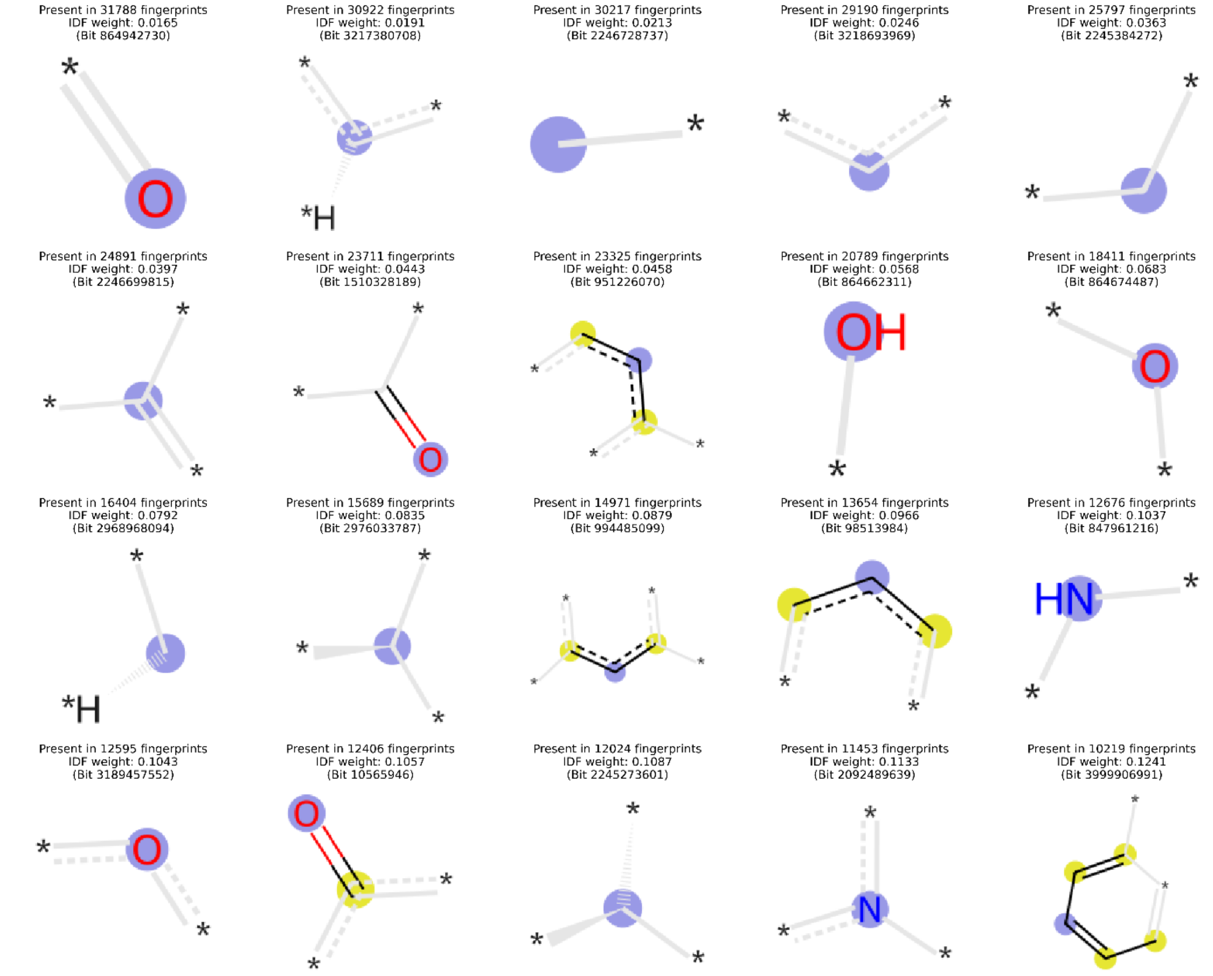

*Figure 12. The 20 most frequently found Morgan-3 fingerprint bits in the **ms2structures dataset**. To avoid bit collisions, sparse Morgan-3 fingerprints were used and bit occurrences were counted. The blue circle marks the center atom.*

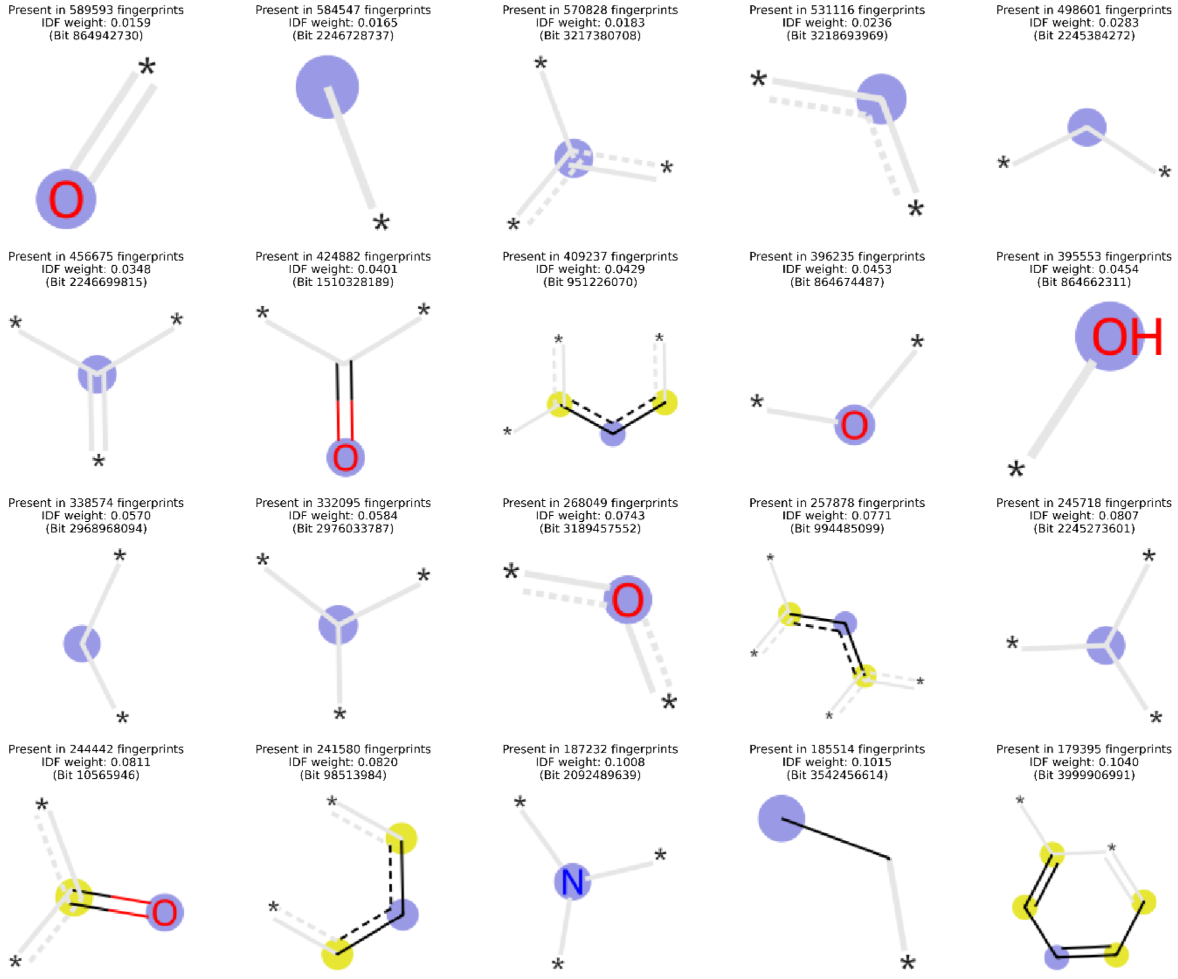

*Figure 13 The 20 most frequently found Morgan-3 fingerprint bits in the **biostructures dataset**. To avoid bit collisions, sparse Morgan-3 fingerprints were used and bit occurrences were counted. The blue circle marks the center atom.*

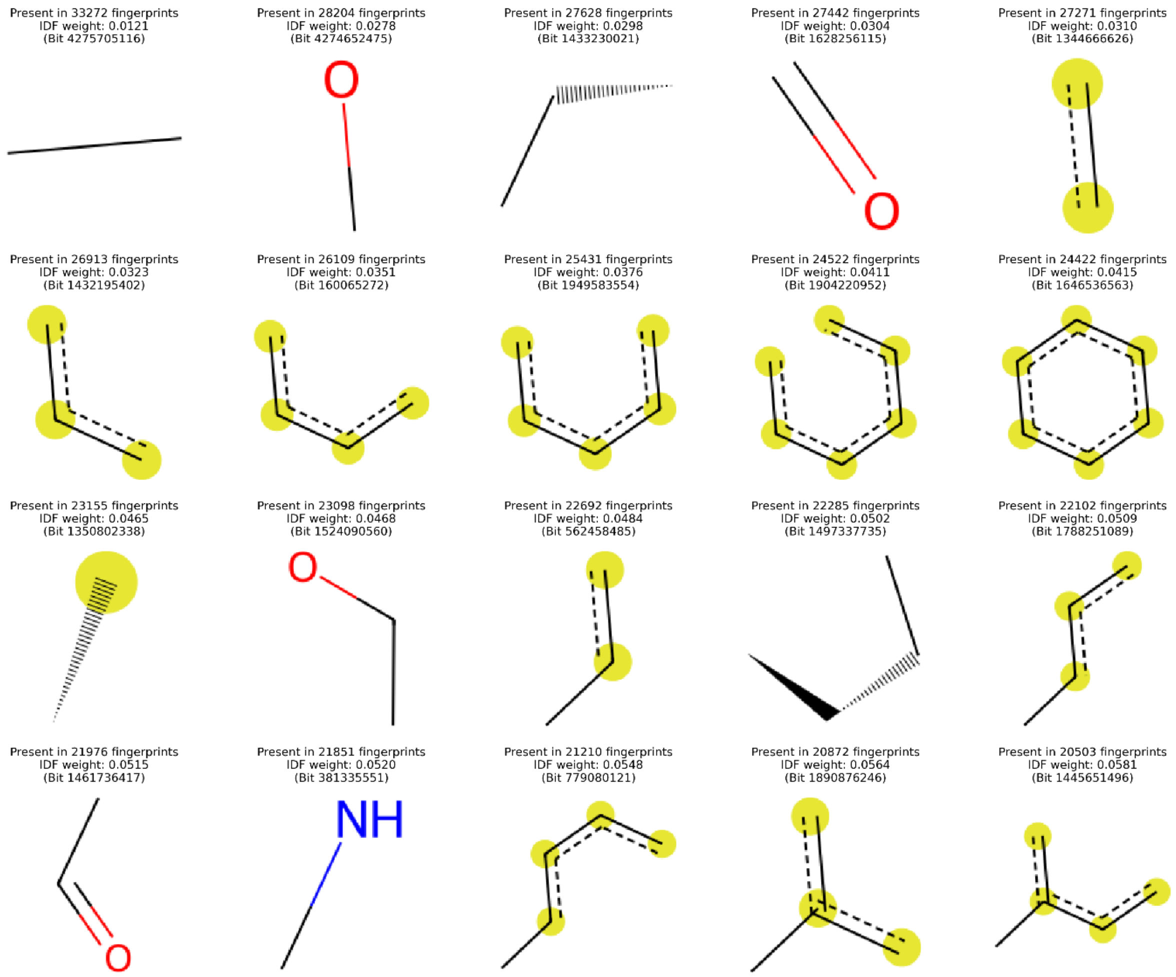

*Figure 14. The 20 most frequently found RDKit fingerprint bits in the **ms2structures dataset**. To avoid bit collisions, sparse RDKit fingerprints were used and bit occurrences were counted. When yellow circles are used, they represent the atoms belonging to the respective paths.*

Additional UMAP visualizations (biostructures dataset, using cuml & cosine)

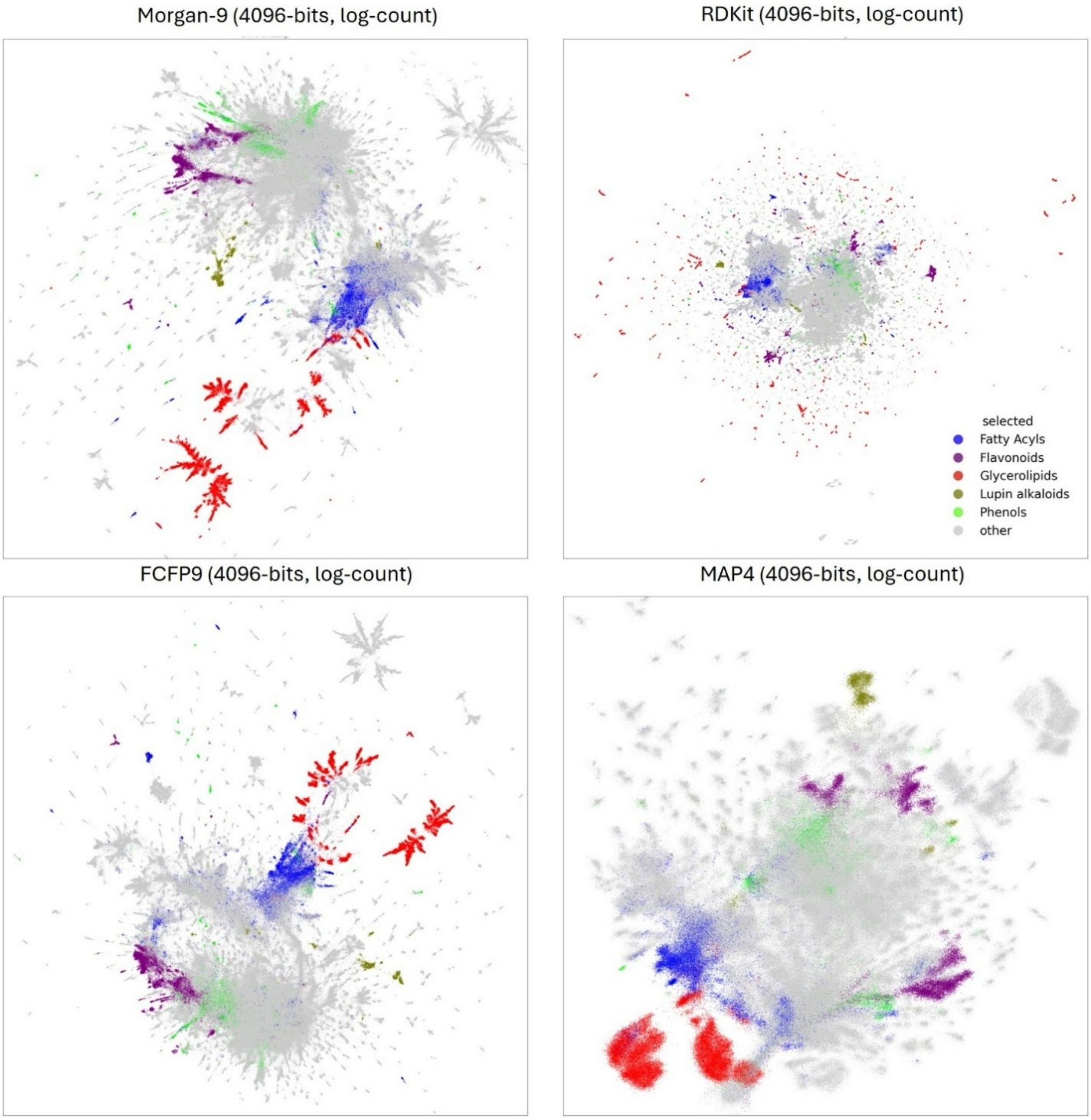

Figure 15 Four examples of UMAP-based chemical space visualizations using the full biostructures dataset. To avoid visual cluttering, only five different chemical classes were selected and colored in the scatter plot. All these UMAP coordinates were computed with chemap using the cuml UMAP implementation which is GPU-optimized. Due to the cuml implementation's restrictions, folded fingerprints had to be used and distances were computed using cosine (instead of Tanimoto as done in all other parts of this work).

Additional UMAP visualizations (ms2structures dataset)

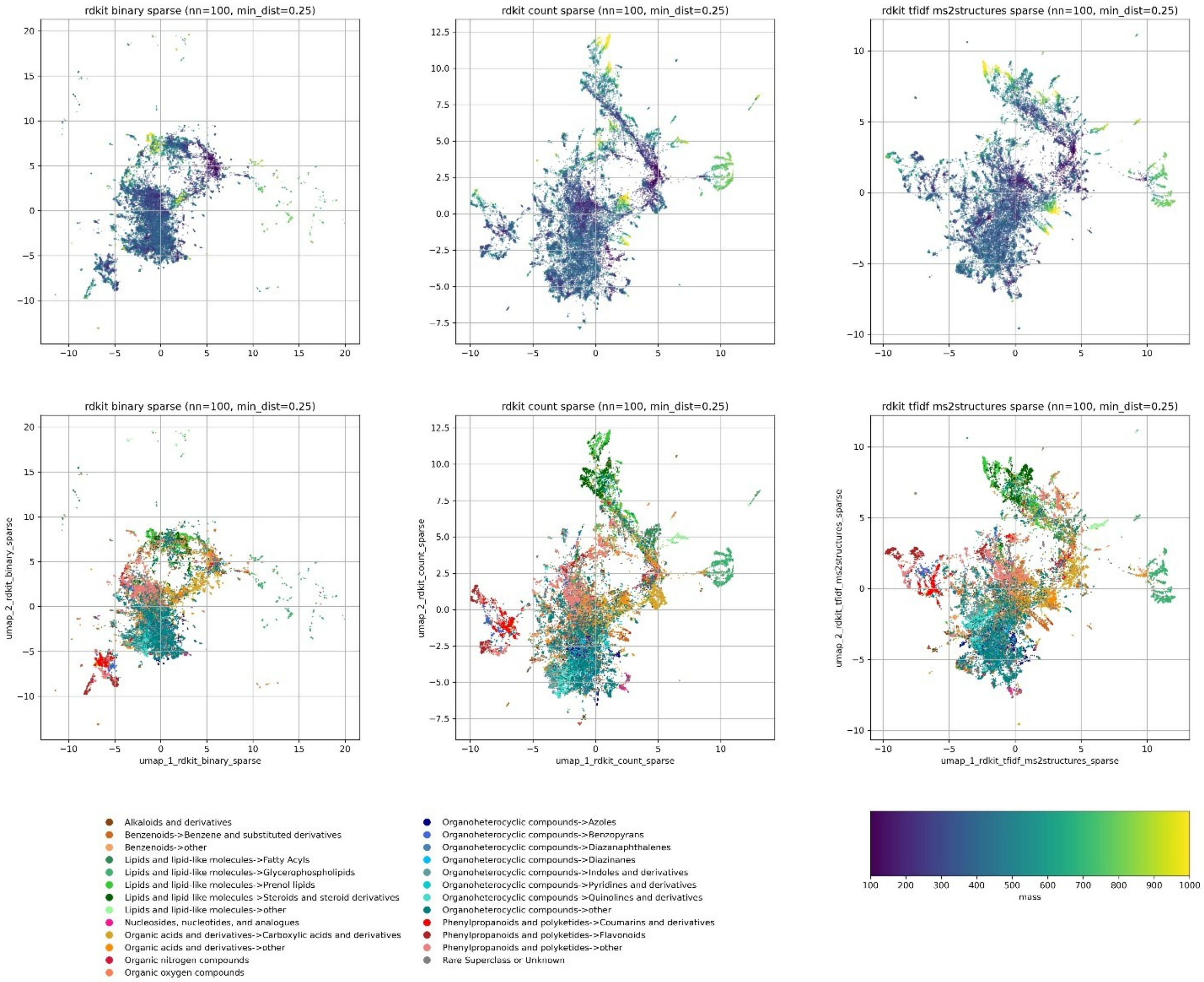

Figure 16 UMAP-based 2D coordinates of all 38,711 compounds in the ms2structures dataset were used to visualize the chemical space of this dataset according to different similarity measures. UMAP was run using the 100 nearest neighbors (and their similarity scores) computed using Tanimoto scores of RDKit binary sparse fingerprints (left panel), of RDKit count sparse fingerprints (center panel) as well as of IDF-weighted RDKit count sparse fingerprints (right panel). In the top row plots every molecule is colored by its molecular mass (in Da), and in the bottom row plots the molecules are colored based on their chemical class according to Classyfire [42] (less common classes were merged to obtain a limited number of categories for better visualization).

References

[1] S. Riniker and G. A. Landrum, “Open-source platform to benchmark fingerprints for ligand-based virtual screening,” *Journal of Cheminformatics*, vol. 5, no. 1, p. 26, May 2013, doi: 10.1186/1758-2946-5-26.

[2] J. Demšar, “Statistical Comparisons of Classifiers over Multiple Data Sets,” *J. Mach. Learn. Res.*, vol. 7, pp. 1–30, Dec. 2006.

[3] A. Capecchi, D. Probst, and J.-L. Reymond, “One molecular fingerprint to rule them all: drugs, biomolecules, and the metabolome,” *Journal of Cheminformatics*, vol. 12, no. 1, p. 43, Jun. 2020, doi: 10.1186/s13321-020-00445-4.

[4] M. Orsi and J.-L. Reymond, “One chiral fingerprint to find them all,” *Journal of Cheminformatics*, vol. 16, no. 1, p. 53, May 2024, doi: 10.1186/s13321-024-00849-6.
